# New records of *Harpiolaisodon* (Chiroptera, Vespertilionidae) from the Chinese mainland

**DOI:** 10.3897/BDJ.12.e120670

**Published:** 2024-03-20

**Authors:** Song Li, Xin Mou, Mengcheng Li, Fengyi Li, Mei Li, Biao Li, Mengjia Li, Xiong Luo, Gábor Csorba, Haochi Kuo

**Affiliations:** 1 Yunnan Key Laboratory of Biodiversity Information, Kunming Institute of Zoology, Chinese Academy of Sciences, Kunming, China Yunnan Key Laboratory of Biodiversity Information, Kunming Institute of Zoology, Chinese Academy of Sciences Kunming China; 2 Kunming Natural History Museum of Zoology, Kunming Institute of Zoology, Chinese Academy of Sciences, Kunming, China Kunming Natural History Museum of Zoology, Kunming Institute of Zoology, Chinese Academy of Sciences Kunming China; 3 State Key Laboratory of Genetic Resources and Evolution, Kunming Institute of Zoology, Chinese Academy of Sciences, Kunming, China State Key Laboratory of Genetic Resources and Evolution, Kunming Institute of Zoology, Chinese Academy of Sciences Kunming China; 4 4-D Genomic Dynamics in Ecology and Evolution, Kunming Institute of Zoology, Chinese Academy of Sciences, Kunming, China 4-D Genomic Dynamics in Ecology and Evolution, Kunming Institute of Zoology, Chinese Academy of Sciences Kunming China; 5 Conservation Area Management Committee of Guanyin Shan Provincial Nature Reserve, Yuanyang, China Conservation Area Management Committee of Guanyin Shan Provincial Nature Reserve Yuanyang China; 6 Hungarian Natural History Museum, Department of Zoology, H-1088 Budapest, Baross u. 13, Hungary Hungarian Natural History Museum, Department of Zoology H-1088 Budapest, Baross u. 13 Hungary; 7 Biodiversity Research Center, Academia Sinica, Taipei, China Biodiversity Research Center, Academia Sinica Taipei China

**Keywords:** distribution, Murininae, tube-nosed bats, Yunnan

## Abstract

**Background:**

The new species, *Harpiolaisodon* Kuo et al., 2006, was described from Taiwan, China. So far, no distribution of this species outside Taiwan has been reported.

**New information:**

During two field investigations of small mammals in Guanyin Mountains Provincial Nature Reserve, Yuanyang, Yunnan, China, in April 2022 and May 2023, five individuals of *Harpiola* were collected in the mid-montane evergreen broad-leaved forest. Our morphological and molecular results reveal that these individuals from the Chinese mainland belong to *Harpiolaisodon*, extending the occurrence of this species well beyond its known distributions in Taiwan, China and Vietnam.

## Introduction

Based on a single specimen from northwest India, initially described as *Murinagrisea* Peters, 1872, Thomas (1915) established *Harpiola* as a distinct genus and the generic diagnostic characters included: the wing membrane is attached to the base of the first toe; the upper incisors, canine and premolars are all subequal in size; the upper canine is slightly higher than the upper anterior premolar; and the lower canine is shorter than the lower posterior premolar.

Tate (1941) accepted *Harpiola* as a valid genus, but others, such as Ellerman and Morrison-Scott (1951), Corbet and Hill (1992), Koopman (1994) and Bates and Harrison (1997), treated it as a subgenus of *Murina*. Based on a second *H.grisea* specimen collected in Mizoram, India, Bhattacharyya (2002) re-established the genus *Harpiola*, although Simmons (2005) still listed it as a subgenus of *Murina*. With the additional 11 *Harpiola* specimens collected from Taiwan, China, Kuo et al. (2006) described *H.isodon* as a new species. By carefully reviewing the full range of documented morphological variation of *Murina* (e.g. Peters (1880), Ognev (1928), Wallin (1969), Maeda (1980), Yoshiyuki (1989), Corbet and Hill (1992)), Kuo et al. (2006) clarified that some morphological characters, such as the attachment point of the plagiopatagium to the hind foot and the reduced canines in both upper and lower toothrows, cannot completely distinguish *Harpiola* and *Murina*. Instead, Kuo et al. (2006) listed valid diagnostic characters between the two genera as follows: 1, the heights of the inner and outer upper incisors are both two-thirds of the upper canine’s height (versus height ratios of up to one-half in *Murina*); 2, the upper toothrow gradually decreases in height from the canine to the first premolar, then to the second premolar, while having these teeth similar in bulk (versus a clearly lower first premolar than the other two teeth in *Murina*); 3, the lower toothrow has the canine, the first premolar and the second premolar similar in both height and bulk (versus a clearly smaller first premolar than the canine in *Murina*); 4, the lower canine is strongly bifid, with the additional cusp well developed (versus a small secondary cingular cusp in *Murina*). In 2006, a *Harpiola* bat was captured in central Vietnam, referred to by Kruskop et al. (2006) as H.cf.isodon, representing the first confirmed record of the species outside of Taiwan, China.

During two field investigations of small mammals in Guanyin Mountains Provincial Nature Reserve, Yuanyang, Yunnan, China, in April 2022 and May 2023, five *Murina*-like bats (n = 2 and 3, respectively) were captured with mist nets, showing the diagnostic characters of *Harpiola* as defined by Kuo et al. (2006). As *Harpiola* has never been reported on the Chinese mainland before, we explored their taxonomic status and carried out comparisons, based on molecular and morphological data as described below.

## Materials and methods

### Sampling

The specimens collected in this study comprise 3 adult males and 2 adult females, according to the development degree of molars and the degree of ossification of forelimb joints. No pregnant females were found. Voucher specimens are deposited in the Kunming Natural History Museum of Zoology, Kunming Institute of Zoology, the Chinese Academy of Sciences (KIZ, CAS), Kunming, China and registered under the numbers KIZ 20220058, 20220089, 20230357, 20230425 and 20230463.

### Molecular analyses

Following the manufacturer’s protocol, total genomic DNA was extracted from muscle samples using the Ezup Column Animal Genomic DNA Purification Kit (Sangon Biotech, China). The complete sequence of the mitochondrial *Cyt b* gene was amplified and sequenced with the primer pair LGL765: GAAAAACCAYCGTTGTWATTCAACT and LGL766: GTTTAATAAGAATYTYAGCTTTGGG (Bickham et al. 1995). Polymerase chain reactions (PCR) were carried out in a total volume of 25 μl containing 1 μl of template DNA, 1 μl of each primer at 10 μM, 1 μl of dNTPmix at 10 mM, 0.2 μl of Taq Plus DNA polymerase (Sangon Biotech, China) at 5 U/μl, 2.5 μl of 10x Taq Buffer and added H_2_O to 25 μl. The PCR thermal profile was: 1, 95°C 5 min for initial denaturation; 2, 94°C 30 sec for denaturation; 3, 63°C (decreases by 0.5°C per cycle) 30 sec for annealing; 4, 72°C 30 sec for extension; 5, 10 cycles of steps 2 to 4; 6, 95°C 30 sec for denaturation; 7, 58°C 30 sec for annealing; 8, 72°C 30 sec for extension; 9, 30 cycles of steps 6 to 8; 10, 72°C 10 min final extension; 11, 4°C for renaturation. PCR products were detected by agarose gel electrophoresis and purified using the SanPrep Column DNA Gel Extraction Kit (Sangon Biotech, China). Finally, purified samples were sequenced by the ABI 3730XL DNA Analyzer (USA) at Sangon Biotech (Shanghai, China). Sequences were edited and assembled using SeqMan in Lasergene 7.1 (DNASTAR Inc., Madison, WI, USA).

The full-length *Cyt b* sequences (1,140 bp) were compared with those from the National Center for Biotechnology Information (NCBI). The sequences were aligned using the default parameters of the ClustalW algorithm in the software MEGA11 (Tamura et al. 2021) and the uncorrected *P*-distances were calculated between pairwise sequences. The pairwise deletion option was used to remove ambiguous positions when calculating genetic distances. The phylogeny of the subfamily Murininae was reconstructed by MEGA11 using the Maximum Likelihood method under a GTR+G+I nucleotide substitution model and the branch support was evaluated by 1,000 bootstrap replicates. ModelFinder (Kalyaanamoorthy et al. 2017) on PhyloSuite v.1.2.2 (Zhang et al. 2020) was used to select the best-fit model (GTR+G+I nucleotide substitution model), based on the Bayesian Information Criterion (BIC) .

### Morphological characteristics

The morphological characters of the five Yunnan specimens were compared with those described for *Harpiolagrisea* and *H.isodon* (Dobson 1878, Thomas 1915, Bhattacharyya 2002, Kuo et al. 2006, Kruskop et al. 2006). We also took external, cranial and dental measurements from our specimens, as described below. Head and body length, tail length, foot length, ear length, forearm length, thumb length, metacarpal lengths and tibia length were measured in the field. The cranial and dental measurements were taken according to Kuo et al. (2006) as follows: total length of skull — from the anterior rim of alveolus of the first upper incisor to the most projecting point of the occipital region; condylobasal length — from the exoccipital condyle to the posterior rim of the alveolus of the first upper incisor; upper canine width— taken across the outer borders of upper canines; upper molar width — taken across the outer crowns of the last upper molars; zygomatic width — the greatest width of the skull across the zygomatic arches; mastoid width — the greatest distance across the mastoid region; postorbital width — the least width of the postorbital constriction; maxillary toothrow length — from the front of the upper canine to the back of the crown of the third molar; upper canine–premolar length — the largest distance from the front of the upper canine to the back of the crown of the posterior premolar; length of mandible — from the anterior rim of the alveolus of the first lower incisor to the most posterior part of the condyle; mandibular toothrow length — from the front of the lower canine to the back of the crown of the third lower molar; lower canine–premolar length — the greatest distance from the front of the lower canine to the back of the crown of the posterior premolar; height of the coronoid process — taken perpendicularly from the extremity of the coronoid process to the ramus mandibulae. Each craniodental measurement was taken three times by Xin Mou with a caliper accurate to 0.01 mm and the average value was reported.

### Ethics statement

Following the Chinese laws and regulations on the protection of wild terrestrial animals (State Council Decree 1992), the field investigations of small mammals in Guanyin Mountains Provincial Nature Reserve and the collection of specimens were approved by the Conservation Area Management Committee of Guanyin Mountains Provincial Nature Reserve and the Ethics Committee of KIZ, CAS.

## Taxon treatments

### Harpiola isodon

Kuo et al., 2006

25A3C971-F1F4-5FD6-8762-52FB29CD42DB

#### Materials

- **Type status:** Other material. **Occurrence:** catalogNumber: KIZ20220058; recordedBy: Song Li et al.; individualCount: 1; sex: male; lifeStage: adult; occurrenceID: C699C5B3-9056-5C72-924C-58E795FC224D; **Taxon:** taxonID: https://www.ncbi.nlm.nih.gov/taxonomy/685777; scientificName: *Harpiolaisodon* (Kuo et al. 2006); kingdom: Animalia; phylum: Chordata; class: Mammalia; order: Chiroptera; family: Vespertilionidae; genus: Harpiola; **Location:** country: China; stateProvince: Yunnan; locality: Guanyinshan Nature Reserve, Mt. Guanyin; verbatimElevation: 2381 m; verbatimCoordinates: 23°1.8'N 102°57‘E; decimalLatitude: 23.03; decimalLongitude: 102.95; georeferenceProtocol: lable; **Event:** eventDate: 24-04-22
- **Type status:** Other material. **Occurrence:** catalogNumber: KIZ20220089; recordedBy: Song Li et al.; individualCount: 1; sex: male; lifeStage: adult; occurrenceID: 47008C54-877D-56A6-AF51-08E0D2BF6EC6; **Taxon:** taxonID: https://www.ncbi.nlm.nih.gov/taxonomy/685777; scientificName: *Harpiolaisodon* (Kuo et al. 2006); kingdom: Animalia; phylum: Chordata; class: Mammalia; order: Chiroptera; family: Vespertilionidae; genus: Harpiola; **Location:** country: China; stateProvince: Yunnan; locality: Guanyinshan Nature Reserve, Mt. Guanyin; verbatimElevation: 2381 m; verbatimCoordinates: 23°1.8'N 102°57‘E; decimalLatitude: 23.03; decimalLongitude: 102.95; georeferenceProtocol: lable; **Event:** eventDate: 24-04-22
- **Type status:** Other material. **Occurrence:** catalogNumber: KIZ20230357; recordedBy: Song Li et al.; individualCount: 1; sex: female; lifeStage: adult; occurrenceID: 4054655C-043F-504B-8EB7-0D8664F2B213; **Taxon:** taxonID: https://www.ncbi.nlm.nih.gov/taxonomy/685777; scientificName: *Harpiolaisodon* (Kuo et al. 2006); kingdom: Animalia; phylum: Chordata; class: Mammalia; order: Chiroptera; family: Vespertilionidae; genus: Harpiola; **Location:** country: China; stateProvince: Yunnan; locality: Guanyinshan Nature Reserve, Mt. Guanyin; verbatimElevation: 2463 m; verbatimCoordinates: 22°59.4'N 102°59.4'E; decimalLatitude: 22.99; decimalLongitude: 102.99; georeferenceProtocol: lable; **Event:** eventDate: 22-05-23
- **Type status:** Other material. **Occurrence:** catalogNumber: KIZ20230425; recordedBy: Song Li et al.; individualCount: 1; sex: male; lifeStage: adult; occurrenceID: A0A6D386-2B57-50FC-A91E-4639AF54638B; **Taxon:** taxonID: https://www.ncbi.nlm.nih.gov/taxonomy/685777; scientificName: *Harpiolaisodon* (Kuo et al. 2006); kingdom: Animalia; phylum: Chordata; class: Mammalia; order: Chiroptera; family: Vespertilionidae; genus: Harpiola; **Location:** country: China; stateProvince: Yunnan; locality: Guanyinshan Nature Reserve, Mt. Guanyin; verbatimElevation: 2463 m; verbatimCoordinates: 22°59.4'N 102°59.4'E; decimalLatitude: 22.99; decimalLongitude: 102.99; georeferenceProtocol: lable; **Event:** eventDate: 22-05-23
- **Type status:** Other material. **Occurrence:** catalogNumber: KIZ20230463; recordedBy: Song Li et al.; individualCount: 1; sex: female; lifeStage: adult; occurrenceID: 5FF3C5EC-DB42-5A77-B109-788CE15590E0; **Taxon:** taxonID: https://www.ncbi.nlm.nih.gov/taxonomy/685777; scientificName: *Harpiolaisodon* (Kuo et al. 2006); kingdom: Animalia; phylum: Chordata; class: Mammalia; order: Chiroptera; family: Vespertilionidae; genus: Harpiola; **Location:** country: China; stateProvince: Yunnan; locality: Guanyinshan Nature Reserve, Mt. Guanyin; verbatimElevation: 2463 m; verbatimCoordinates: 22°59.4'N 102°59.4'E; decimalLatitude: 22.99; decimalLongitude: 102.99; georeferenceProtocol: lable; **Event:** eventDate: 22-05-23

#### Description

**Figure 1.**
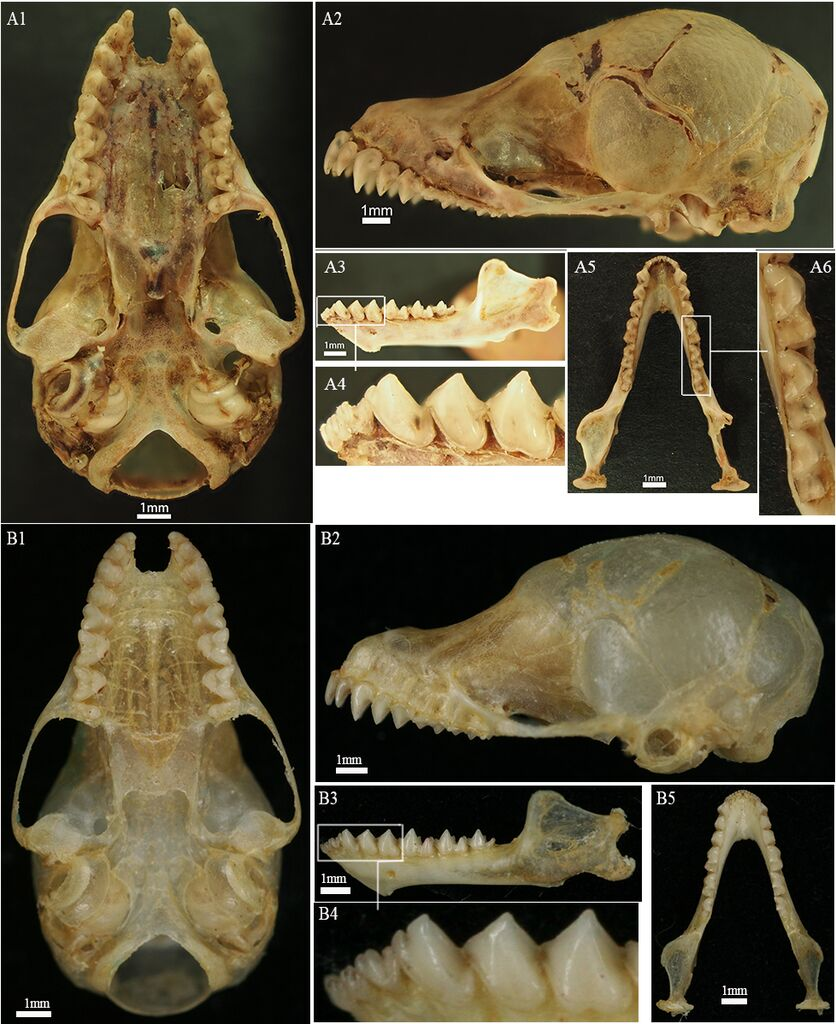
Skull of *Harpiolaisodon*. **A** is from Yunnan; **B** is from Taiwan. 1 = ventral view of skull; and 2 = lateral view of skull; 3 = lateral view of mandible; 4 = details of the anterior lower dentition; 5 = occlusal view of mandible; and 6 = details of the occlusal view of lower molars.

The five Chinese mainland specimens show the distinguishing dental characters of *Harpiola* as defined above (Fig. 1A2, A3 and A4).

##### Body

**Figure 2.**
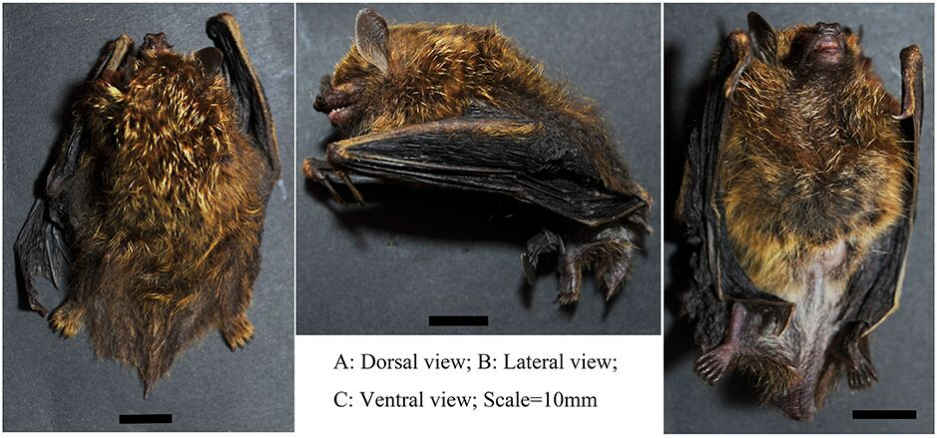
Individual of *Harpiolaisodon* (KIZ20230058) from Yunnan.

A medium-sized bat with the forearm length ranging from 32.33–36.12 mm (Table 1). The wing membrane is attached to the base of the first toe and the interfemoral membrane is attached to the end of the tibia (Fig. 2A and C). The nostril is slightly tubular, but not very prominent (Fig. 2A and C). The ear is small, with the tip slightly blunt and rounded and the tragus is slender and slightly curved, with its length over half that of the ear. The thumb, equipped with a curved, sharp claw, has a length nearly 20% of the forearm length (Fig. 2B and C; Table 1). The third, fourth and fifth metacarpals have about the same length (Table 1). The foot length is about 80% of the tibia length (Table 1). The tail, which is about 70% of the head and body length (Table 1), has its very end excluded from the interfemoral membrane (Fig. 2A).

##### Fur

The face is brownish-black at the snout, becoming paler at the cheeks and around the eyes. The forehead is brown and the chin is brownish-black. Ears are naked, dark brown. The fur is soft and dense, with individual hairs on both dorsal and ventral sides yellowish-brown at their upper parts and dark brown at the bases (a little grey). Both dorsal and ventral furs have golden-tipped hairs (Fig. 2) and there are more such hairs on the back than on the abdomen. On the dorsum, the golden-tipped hairs extend from the top of the head to the back of the interfemoral membrane, where they become sparser. There are yellowish-brown hairs covering the back of the forearm, the back of the thumb and the back of the toes. The golden-tipped hairs on the ventrum are mainly concentrated on the chest. The anal area is brownish-grey. The dorsal and ventral surfaces of the interfemoral membrane are both coated, with thicker, dark brown hairs on the dorsal surface and sparser, light brownish-grey hairs (slightly yellow) on the ventral surface. The foot soles are naked, lacking any sole pad (Fig. 2C). The wing membrane is brownish-black in colour and the area near the side of the body is sparsely coated, otherwise is bare (Fig. 2).

##### Skull

In lateral view, the skull shows a gradually rising curve from the front of the rostrum to the back of the frontal area, with the centre of the forehead slightly depressed. The braincase is rounded, the sagittal crest is absent and the lambdoid crest is moderately developed (Fig. 1A2). The zygomatic arches are weak and slender, showing the most outward expanded points at their posterior ends. The tympanic bullae are relatively small and the foramen magnum is relatively large (Fig. 1, A1).

##### Dentition

Dental formula: I \begin{varwidth}{50in}\begin{equation*}
            - 2 3\over 1 2 3
        \end{equation*}\end{varwidth} C \begin{varwidth}{50in}\begin{equation*}
            1\over1
        \end{equation*}\end{varwidth} PM \begin{varwidth}{50in}\begin{equation*}
            -2-4\over-2-4
        \end{equation*}\end{varwidth} M \begin{varwidth}{50in}\begin{equation*}
            1 2 3\over 1 2 3
        \end{equation*}\end{varwidth}\begin{varwidth}{50in}\begin{equation*}
             
        \end{equation*}\end{varwidth}= 34. Upper incisors are large, their heights are more than two-thirds that of C^1^ and the outer incisor (I^3^) is slightly lower than the inner incisor (I^2^) (Fig. 1, A2). C^1^, PM^2^ and PM^4^ are gradually decreasing in height and their base sizes are similar. Amongst the three upper molars, the last one (M^3^) has a crown area only half that of each anterior one (Fig. 1, A1). The lower toothrows contain three lower incisors on each side, with their heights gradually increasing from the inner (I_1_) to the outer one (I_3_). Each lower incisor has three cusps, with a marked depression between the outermost cusp and the middle cusp. The lower canine (C_1_), with two blunt cusps, is about the same height as the anterior and posterior lower premolars (PM_2_ and PM_4_, respectively) (Fig. 1, A4). The crown areas of M_1_ and M_2_ are slightly larger than M_3_; the postcristids are well developed and the hypoconids are more prominent than the hypoconulids (Fig. 1, A6).

## Discussion

### Morphological traits

The two species of *Harpiola*, *H.isodon* and *H.grisea*, differ from each other in the following characters: the PM^4^ is wider than long in *H.isodon* (versus as wide as long in *H.grisea*); both M^1^ and M^2^ have the mesostyles present in *H.isodon* (versus M^2^ lacking mesostyle in *H.grisea*); M^1^ of *H.isodon* has a post-cingular platform (versus no post-cingular platform in *H.grisea*) (Kuo et al. 2006). The five Chinese mainland *Harpiola* specimens showed these dental characters in line with *H.isodon*. We noted that the Chinese mainland taxon had a range of maxillary toothrow length surpassing that of the Taiwanese *H.isodon* provided in Kuo et al. (2006) (Table 1). Nevertheless, we should warn that the measurements were taken by different people across the two studies and those taken by the same investigator are warranted in the future for a valid evaluation of the morphometric differences between the two taxa.

### Genetic distances and phylogenetic relationships

We compared the *Cyt b* sequences of our five specimens with those downloaded from NCBI for 19 Vespertilionids, including *Harpiolaisodon* from Taiwan, *Harpiocephalusharpia*, 15 *Murina*, one *Kerivoula* and one *Myotis* species (Table 2). The novel sequences are deposited in the NCBI GenBank database under accession numbers PP476123 (KIZ20220058), PP476124 (KIZ20220089), PP476125 (KIZ20230357), PP476126 (KIZ20230425) and PP476127 (KIZ20230463).

**Figure 3.**
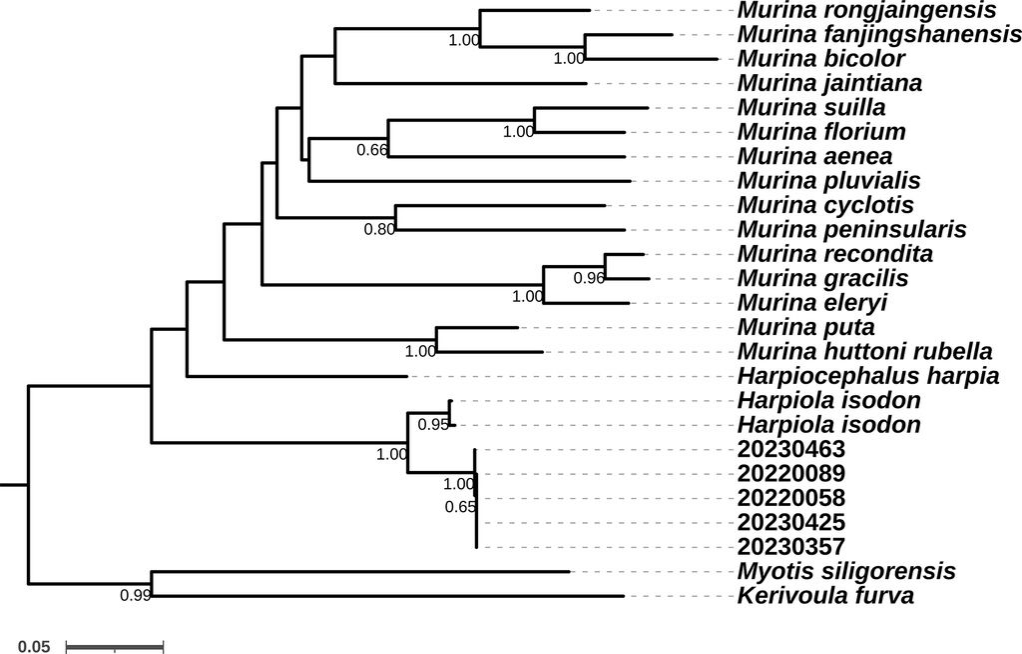
Maximum Likelihood phylogenetic reconstruction of 25 Vespertilionidae samples using an 1140 bp alignment of the mitochondrial *cyt b* gene. Bootstrap (BS) values are indicated adjacent to nodes (nodes with BS < 0.50 are not labelled).

Based on the reconstructed phylogenetic tree, the Chinese mainland *Harpiola* formed a monophyletic group with *H.isodon* (Fig. 3), which indicates a close relationship between them. However, the two taxa showed genetic distances of 4.8–5.1% between each other, which exceeded the difference between *Murinarecondita* and *Murinagracilis* (3.7%) and was not much lower than those between *Murinasuilla* and *Murinaflorium* (7.7%), *Murinagracilis* and *Murinaeleryi* (7.7%) and *Murinaputa* and *Murinahuttonirubella* (7.2%) (Suppl. material 1). Thus, there are substantial sequence differences in the studied uniparentally inherited gene between Taiwanese and mainland *Harpiola* bats, but the taxonomic interpretation of these differences would be premature and additional material and nuclear genes should be included in subsequent analyses.

With a synthetic consideration of morphological and molecular evidence, we refer to the specimens from the Guanyin Mountains, Yuanyang, Yunnan as the first records of *H.isodon* on the Chinese mainland.

### Ecological notes

**Figure 4.**
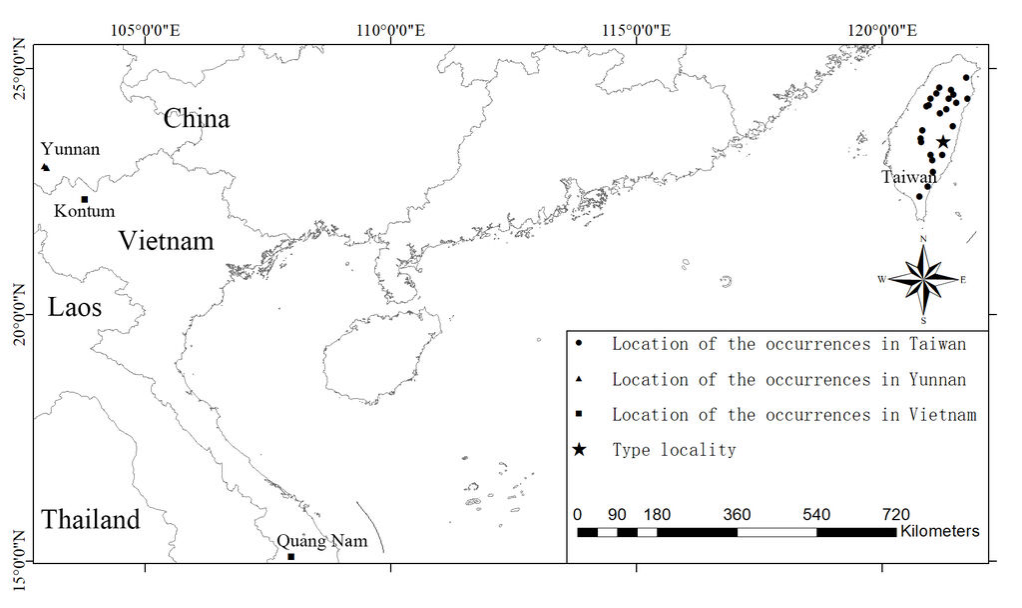
Distribution map of *Harpiolaisodon*. Coordinate information is derived from Kuo et al. (2006), Kruskop et al. (2006) and the Global Biodiversity Information Facility (Anonymous 2024).

The specimens were captured at two mountain sites (23.03N, 102.95E and 22.99N, 102.99E) with mist nets. Their locations and the distribution map of *Harpiolaisodon* are shown in Fig. 4. The habitat is a mid-mountain evergreen broad-leaved forest at elevations of 2,381 m and 2,463 m, respectively. The canopy is well closed and the forest is rich in shrubs and has small streams. There are no caves in this area, but there are many large trees with tree holes. We speculate that this insect-eating bat mainly roosts in tree cavities or under the dense canopy during the daytime.

## Supplementary Material

XML Treatment for Harpiola isodon

E674AD1F-4E4C-5419-BBB1-894D8B08628C 10.3897/BDJ.12.e120670.suppl1 Supplementary material 1 Uncorrected pairwise genetic *P*-distance Data type pairwise genetic *P*-distance Brief description Uncorrected pairwise genetic *P*-distance (%) amongst the species on 1140 bp of mitochondrial Cyt b. File: oo_977044.docx https://binary.pensoft.net/file/977044 Xin Mou

## Figures and Tables

**Table 1.** External and craniodental measurements of *Harpiola* species (mm) (*the parameter shows obvious difference between specimens of Yunnan and Taiwan).

Parameter	*H.isodon* Yunnan, China, This study		*H.isodon* Taiwan, China, (Kuo et al. 2006)	H.cf.isodon, Vietnam, (Kruskop et al. 2006）	*H.grisea*, Mizoram, India, (Bhattacharyya 2002)
	Range (n=5)	Mean (n=5)			
Head and body length	44.69-46.50	45.47	-	-	42.7
Tail length	30.03-36.95	31.86	-	-	27.5
Foot length	9.92-10.94	10.26	-	-	8.20
Ear length	12.42-15.17	14.08	12.50-13.00	11.80	12.10
Forearm length	32.33-36.12	34.61	31.00-35.60	31.70	32.40
Thumb length	6.42-6.97	6.65		-	-
The third metacarpal length	29.21-32.21	30.62		-	-
The fourth metacarpal length	29.01-31.95	30.34		-	-
The fifth metacarpal length	28.91-31.85	30.21		-	-
Tibia length	12.14-12.77	12.37		-	14.80
Total length of skull	15.54-16.27	15.87	14.76-16.48	15.41	16.40
Condylobasal length	14.12-15.17	14.83	13.74-14.87	14.49	-
Upper canine width	3.82-4.10	3.95	3.65-4.02	3.98	3.70
Upper molar width	4.80-5.74	5.36	4.90-5.53	5.32	5.50
Zygomatic width	8.72-9.75	9.32	8.43-9.35	8.94	9.40
Mastoid width	7.77-8.21	8.04	7.29-7.96	7.68	-
Postorbital width	4.80-5.03	4.91	4.52-4.84	4.47	4.30
Maxillary toothrow length*	**5.74-5.87**	**5.82**	**4.97-5.63**	5.39	5.30
Upper canine–premolar length	2.35-2.64	2.52	2.22-2.73	-	-
Length of mandible	10.57-11.27	10.92	10.15-11.32	10.80	10.60
Mandibular toothrow length	5.63-6.08	5.88	5.35-5.90	5.68	5.70
Lower canine–premolar length	2.31-2.47	2.40	2.02-2.44	-	-
Height of the coronoid process	3.33-3.73	3.47	3.36-3.98	-	-

**Table 2.** Additional samples and their GenBank accession numbers used in the phylogenetic reconstruction.

species	GenBank	species	GenBank
*Harpiolaisodon*	GQ168914	*Murinaflorium*	GQ168902
*Harpiolaisodon*	GQ168920	*Murinagracilis*	GQ168900
*Harpiocephalusharpia*	GQ168923	*Murinarecondita*	KJ198270
*Murinaeleryi*	GQ168908	*Murinabicolor*	JQ044696
*Murinacyclotis*	MK747248	*Murinafanjingshanensis*	KT180333
*Murinasuilla*	GQ168905	*Murinarongjiangensis*	MF359930
*Murinaputa*	GQ168901	*Murinapluvialis*	JQ044689
*Murinaaenea*	GQ168906	*Murinapeninsularis*	GQ168911
*Murinahuttonirubella*	KU521385	*Myotissiligorensis*	FJ215679
*Murinajaintiana*	JQ044690	*Kerivoulafurva*	MH208497
