# Supplementary material for: New records of Harpiolaisodon (Chiroptera, Vespertilionidae) from the Chinese mainland
Source: Biodivers Data J. 2024 Mar 20;12:e120670. doi: 10.3897/BDJ.12.e120670 (PMC10973766; doi:10.3897/BDJ.12.e120670)
Supplement: Supplementary material 1 — Uncorrected pairwise genetic P-distance [file bdj-12-e120670-s001.docx]

Uncorrected pairwise genetic P-distance(%) among the species on 1140bp of mitochondrial Cyt b.

|  | 1 | 2 | 3 | 4 | 5 | 6 | 7 | 8 | 9 | 10 | 11 | 12 | 13 | 14 | 15 | 16 | 17 | 18 | 19 | 20 | 21 | 22 | 23 | 24 |
| --- | --- | --- | --- | --- | --- | --- | --- | --- | --- | --- | --- | --- | --- | --- | --- | --- | --- | --- | --- | --- | --- | --- | --- | --- |
| 1 |  |  |  |  |  |  |  |  |  |  |  |  |  |  |  |  |  |  |  |  |  |  |  |  |
| 2 | 0.0 |  |  |  |  |  |  |  |  |  |  |  |  |  |  |  |  |  |  |  |  |  |  |  |
| 3 | 0.0 | 0.0 |  |  |  |  |  |  |  |  |  |  |  |  |  |  |  |  |  |  |  |  |  |  |
| 4 | 0.0 | 0.0 | 0.0 |  |  |  |  |  |  |  |  |  |  |  |  |  |  |  |  |  |  |  |  |  |
| 5 | 0.1 | 0.1 | 0.1 | 0.1 |  |  |  |  |  |  |  |  |  |  |  |  |  |  |  |  |  |  |  |  |
| 6 | **5.1** | **5.1** | **5.1** | **5.1** | **5.0** |  |  |  |  |  |  |  |  |  |  |  |  |  |  |  |  |  |  |  |
| 7 | **4.9** | **4.9** | **4.9** | **4.9** | **4.8** | 0.4 |  |  |  |  |  |  |  |  |  |  |  |  |  |  |  |  |  |  |
| 8 | 18.6 | 18.6 | 18.6 | 18.6 | 18.5 | 17.7 | 17.7 |  |  |  |  |  |  |  |  |  |  |  |  |  |  |  |  |  |
| 9 | 16.3 | 16.3 | 16.3 | 16.3 | 16.2 | 14.7 | 14.9 | 17.0 |  |  |  |  |  |  |  |  |  |  |  |  |  |  |  |  |
| 10 | 16.6 | 16.6 | 16.6 | 16.6 | 16.5 | 15.3 | 15.1 | 17.4 | 10.1 |  |  |  |  |  |  |  |  |  |  |  |  |  |  |  |
| 11 | 16.9 | 16.9 | 16.9 | 16.9 | 16.8 | 16.4 | 16.4 | 16.8 | 11.2 | 8.3 |  |  |  |  |  |  |  |  |  |  |  |  |  |  |
| 12 | 16.2 | 16.2 | 16.2 | 16.2 | 16.1 | 15.9 | 15.7 | 16.7 | 13.8 | 14.6 | 15.3 |  |  |  |  |  |  |  |  |  |  |  |  |  |
| 13 | 18.0 | 18.0 | 18.0 | 18.0 | 17.9 | 17.6 | 18.0 | 18.3 | 15.6 | 16.6 | 18.0 | 14.9 |  |  |  |  |  |  |  |  |  |  |  |  |
| 14 | 16.8 | 16.8 | 16.8 | 16.8 | 16.7 | 16.7 | 16.7 | 16.2 | 14.6 | 15.5 | 15.7 | 13.4 | ***7.7*** |  |  |  |  |  |  |  |  |  |  |  |
| 15 | 15.8 | 15.8 | 15.8 | 15.8 | 15.7 | 16.0 | 16.0 | 18.0 | 16.3 | 15.9 | 16.6 | 15.6 | 12.9 | 13.7 |  |  |  |  |  |  |  |  |  |  |
| 16 | 16.9 | 16.9 | 16.9 | 16.9 | 16.8 | 16.2 | 16.3 | 17.0 | 15.1 | 16.1 | 16.5 | 15.3 | 15.3 | 16.0 | 15.3 |  |  |  |  |  |  |  |  |  |
| 17 | 16.6 | 16.6 | 16.6 | 16.6 | 16.5 | 16.2 | 16.4 | 16.2 | 15.6 | 17.6 | 17.3 | 14.9 | 14.6 | 16.0 | 15.2 | 14.9 |  |  |  |  |  |  |  |  |
| 18 | 15.0 | 15.0 | 15.0 | 15.0 | 14.9 | 14.5 | 14.7 | 17.0 | 15.3 | 16.4 | 16.0 | 15.9 | 16.3 | 17.0 | 15.0 | 15.4 | 12.8 |  |  |  |  |  |  |  |
| 19 | 16.8 | 16.8 | 16.8 | 16.8 | 16.7 | 17.2 | 17.4 | 18.8 | 15.9 | 17.4 | 17.8 | 15.8 | 16.1 | 16.1 | 15.3 | 16.2 | 16.9 | 18.1 |  |  |  |  |  |  |
| 20 | 17.2 | 17.2 | 17.2 | 17.2 | 17.1 | 17.2 | 17.2 | 18.5 | 16.2 | 18.3 | 18.2 | 16.5 | 17.0 | 16.4 | 16.1 | 16.3 | 16.5 | 18.4 | ***3.7*** |  |  |  |  |  |
| 21 | 15.8 | 15.8 | 15.8 | 15.8 | 15.7 | 16.1 | 16.5 | 18.1 | 16.5 | 17.7 | 17.4 | 16.6 | 16.5 | 15.6 | 15.0 | 17.1 | 16.5 | 16.4 | 7.4 | ***7.7*** |  |  |  |  |
| 22 | 16.7 | 16.7 | 16.7 | 16.7 | 16.7 | 15.7 | 15.7 | 16.4 | 14.2 | 16.1 | 15.3 | 16.8 | 17.8 | 16.9 | 16.9 | 17.5 | 16.2 | 16.0 | 15.2 | 14.7 | 15.9 |  |  |  |
| 23 | 15.6 | 15.6 | 15.6 | 15.6 | 15.6 | 16.1 | 16.1 | 16.7 | 15.8 | 16.7 | 16.5 | 16.8 | 17.0 | 17.2 | 16.4 | 16.9 | 16.1 | 16.8 | 15.6 | 15.3 | 16.5 | ***7.2*** |  |  |
| 24 | 18.2 | 18.2 | 18.2 | 18.2 | 18.1 | 18.3 | 18.5 | 20.3 | 18.5 | 19.1 | 19.6 | 19.3 | 18.3 | 18.3 | 19.2 | 19.0 | 17.7 | 18.3 | 18.7 | 19.5 | 19.2 | 20.4 | 20.9 |  |
| 25 | 18.9 | 18.9 | 18.9 | 18.9 | 18.8 | 18.6 | 18.5 | 20.3 | 18.9 | 20.4 | 19.9 | 19.2 | 19.2 | 19.2 | 19.7 | 19.8 | 18.8 | 19.4 | 19.7 | 20.5 | 19.9 | 19.2 | 19.5 | 17.7 |

1-5-*Harpiola isodon*, Yunnan, 6-7-*Harpiola isodon*, Taiwan, 8-*Harpiocephalus harpia*, 9-*Murina rongjaingensis*, 10-*Murina fanjingshanensis*, 11-*Murina bicolor*, 12-*Murina jaintiana*, 13-*Murina suilla*, 14-*Murina florium*, 15-*Murina aenea*, 16-*Murina pluvialis*, 17-*Murina cyclotis*, 18-*Murina peninsularis*, 19-*Murina recondita*, 20-*Murina gracilis*, 21-*Murina eleryi*, 22-*Murina puta*, 23-*Murina huttoni rubella*, 24-*Myotis siligorensis*, 25-*Kerivoula furva*.
